# The Enhanced Recovery After Surgery Pathway Is Safe, Feasible and Cost-Effective in Delayed Graft Function After Kidney Transplant

**DOI:** 10.3390/jcm14072387

**Published:** 2025-03-31

**Authors:** Francesca Romano, Roberta Angelico, Luca Toti, Michela Orsi, Valentina Enrica Marsella, Tommaso Maria Manzia, Leonardo Emberti Gialloreti, Giuseppe Tisone

**Affiliations:** 1 HPB and Transplant Unit, Department of Surgical Sciences, University of Rome Tor Vergata, 00133 Rome, Italy; francesca.romano@ptvonline.it (F.R.); toti@med.uniroma2.it (L.T.); michela.orsi@ptvonline.it (M.O.); valentinaenrica.marsella@ptvonline.it (V.E.M.); manzia@med.uniroma2.it (T.M.M.); tisone@med.uniroma2.it (G.T.); 2 Department of Biomedicine and Prevention, University of Rome Tor Vergata, Via Montpellier 1, 00133 Rome, Italy; leonardo.emberti.gialloreti@uniroma2.it

**Keywords:** kidney transplantation, delayed graft function, enhanced recovery after surgery protocols, healthcare costs, cost-effectiveness

## Abstract

**Background/Objectives:** Enhanced recovery after surgery (ERAS) pathways are still underutilized in kidney transplantation (KT), and their feasibility after delayed graft function (DGF) is unknown. We aimed to evaluate safety and cost savings after ERAS implementation in KT recipients with DGF. **Methods:** A retrospective analysis of KT recipients enrolled in the ERAS program with DGF (≥1 dialytic treatment during the first postoperative week or creatinine≥ 2.5 mg/dL on postoperative day 10) between 2010 and 2019 was performed. Recipient, donor, and transplant data, outcomes, and 1-year post-KT costs were collected, comparing recipients within the ERAS target (≤5 days, “early discharge group”) to those discharged later (>5 days, “late discharge group”). **Results:** Out of 170 KT recipients with DGF, 33 (19.4%) were in the “early discharge group” and 137 (80.5%) in the “late discharge group”. Recipient, donor, and transplant characteristics were similar in the two groups. The length of hospital stay (LOS) of the “early discharge group” was significantly shorter, with fewer in-hospital dialysis sessions (*p* < 0.001) compared to the “late discharge group”. One year post-KT, no significant differences were observed in postoperative complications, readmissions, or number of outpatient visits. Five-year graft and patient survival along with five-year graft function were similar between the two cohorts. First-year costs were significantly higher in the “late discharge group” (*p* < 0.001), with a median excess cost (Δ) of EUR 4515.76/patient. Factors influencing first-year costs post-KT were LOS for KT, recipient age, and use of expanded-criteria grafts. **Conclusions:** The ERAS approach is safe in KT recipients with DGF and allows for economic savings, while its implementation does not cause worse clinical outcomes in recipients.

## 1. Introduction

Enhanced recovery after surgery (ERAS) programs have gained widespread diffusion among different surgical fields as they provide advantages in terms of rapid postoperative recovery and early resumption of daily activities while reducing lengths of hospital stay (LOS) and healthcare costs [1,2]. ERAS programs initially found their fertile ground for implementation in elective settings, such as colorectal surgery [3,4]. Later, ERAS pathways have been progressively applied in most surgical specialties with standardized protocols, including well-structured pre-, intra-, and postoperative steps [1,2,3,4,5], individualized for patient populations [2].

In kidney transplantation (KT), ERAS protocols have reached a lower spread due to multiple factors. Firstly, candidates for KT have been traditionally considered frail individuals due to their challenging anesthesiologic management, cardiovascular comorbidities, long-standing dialysis therapy, and postoperative immunosuppressive status [6,7,8]. Secondly, delayed graft function (DGF) may raise an even greater concern in implementing ERAS protocols after KT. After DGF occurrence, composite postoperative in-hospital management and, in the case of a patient being discharged early, the need for a close collaboration between the transplant center and the peripheral dialytic center entail logistical and economic efforts from both patient and clinician perspectives. Although several strategies have been implemented to reduce the incidence of DGF [9], such as the use of machine perfusion [10], this aspect becomes particularly relevant considering the expected increase in the incidence of this condition after transplantation. The growing use of grafts from expanded-criteria donors (ECD), implemented to address the shortage of grafts, and the long waiting list for KT candidates may further exacerbate this scenario [11,12,13,14,15].

Although previous studies have suggested that ERAS implementation in KT may have led to several advantages in terms of reduction in LOS, postoperative complication rates, and cost-effectiveness [16,17,18,19,20,21], there is still a lack of knowledge regarding the safety and applicability of ERAS protocols in KT recipients who develop DGF. Moreover, few studies analyzed the impact of DGF after KT on healthcare costs [22,23], but this analysis has never been conducted within ERAS programs.

In our center, the ERAS protocol for KT recipients has been implemented for the last decade, and we recently demonstrated that the ERAS pathway is safe and feasible in all patients receiving KT, allowing early discharge and clinical benefits [21]. We also identified that the failure of an early discharge, within the ERAS target, is not related to the recipient’s clinical outcomes.

The primary aim of the present study was to evaluate the feasibility and safety of ERAS pathways’ implementation specifically in KT recipients who develop DGF, analyzing the postoperative outcomes within one year after transplantation and identifying the predictive factors of prolonged hospitalization (beyond five days after KT). Our secondary aim was to calculate the first-year post-KT healthcare costs when DGF occurs within the “ERAS target” (LOS ≤ 5 days after transplantation) or not.

## 2. Materials and Methods

### 2.1. Study Design

We report a single-center retrospective study that included all KT recipients who developed DGF after transplantation, between January 2010 and December 2019, with at least 3 months of follow-up. All KT recipients were enrolled in the ERAS protocol and had been analyzed in a previous study [21] approved by our Ethical Committee and registered under research registry 37.21; the data from patients with DGF were subsequently collected. Recipients of simultaneous liver–kidney or pancreas–kidney transplantation, patients with a follow-up of less than 3 months after KT, and those under the age of 18 at the time of transplantation were excluded from the study. DGF was defined as the requirement of at least one dialytic treatment during the first postoperative week or increased levels of creatinine above 2.5 mg/dL on postoperative day 10 [24,25].

### 2.2. Data Collection

Data were collected retrospectively. Recipient data at the time of transplantation [age, gender, body mass index (BMI), causes of end-stage kidney disease (ESKD), waiting list time for KT, comorbidities], donor variables [deceased or living donors, age, cause of death, comorbidities, and if the graft was procured from ECD], and graft or transplant features [single or dual, re-transplantation, sequential KT, pre-implant renal biopsy, and cold ischemia time (CIT)] were examined. ECD was defined as donor age ≥ 60 years or donor age ≥ 50 years with at least two of the following donor variables: arterial hypertension on chronic medical treatment, death from cerebrovascular cause or pre-procurement creatinine serum level ≥ 1.5 mg/dL [21,26]. When executed before implantation, kidney biopsy was evaluated using the Italian necro-kidney score, which is based on the percentage of sclerosed glomeruli (grade: 0–3), tubular atrophy (grade: 0–3), interstitial fibrosis (grade: 0–3), and atherosclerosis (grade: 0–3), resulting in a total score from 0 to 12 [21,27]. Pre-implantation graft biopsy was executed in each case of donors over 65 years, in line with the Italian national rules. In the first few years of the study (2010–2012), kidneys with a score of 3 or lower were allocated as single transplants, while dual transplantations relied on grafts with a score of 4 and 5, with the assumption that the number of functioning nephrons in the two grafts reflected the number of one optimal kidney. From 2013 onwards, grafts with a score of 4 were also used for single KT; kidneys with a score of 5 were managed as single or dual transplants based on the prevalent histological element composing the score [21,28].

### 2.3. ERAS Pathway

**Figure 1:**
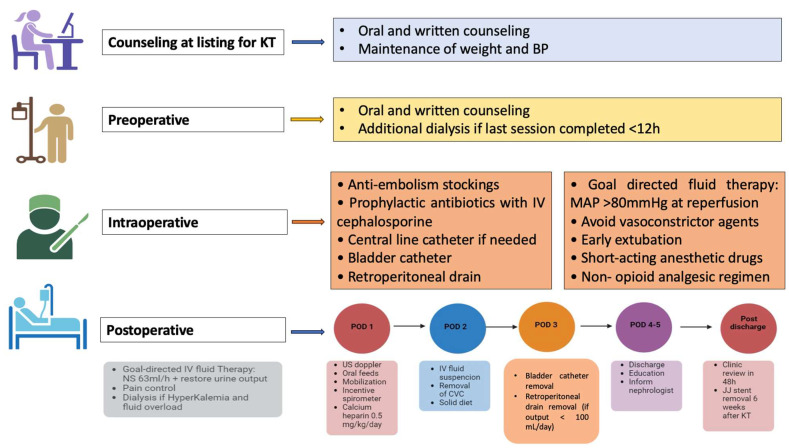
Flow-chart of the enhanced recovery after surgery pathway. Abbreviations: kidney transplant (KT); blood pressure (BP); mean arterial pressure (MAP); intravenous (IV); 0.9% saline fluid solution (NS); postoperative day (POD); ultrasound (US); and central venous catheter (CVC).

From January 2010 onwards, ERAS pathways have been adopted in patients receiving KT in our transplant unit. As described in our previous paper, our ERAS protocol consisted of four stages. The program focused on (1) counseling at the time of the listing for KT, (2) the preoperative period, (3) the intraoperative step, and (4) the postoperative phase [21]. The aim of our program was a rapid postsurgical recovery and discharge home by day 5 after transplantation, irrespective of the resumption of the graft’s function (Figure 1).

### 2.4. Immunosuppressive Therapy

Induction therapy with basiliximab (20 mg intraoperatively and on the fourth postoperative day) or antithymocyte globulin (three doses of 1.5 mg/kg for each dose) was administered according to the preoperative panel-reactive antibody (PRA) score. Maintenance immunosuppressive therapy relied on tacrolimus once daily (0.15 mg/kg/day), mycophenolate mofetil (500–1500 mg/day), or sodium (360–1440 mg/day) and steroids (20 mg/day with tapering of the dosage up to 5 mg/day within 1 month). Tacrolimus trough levels were adopted according to the time elapsed from transplantation (7–9 ng/mL in the first month after KT, 6–8 mg/mL within six months, and 5–6 ng/mL thereafter). In some cases, changes in immunosuppression, primarily the reduction in calcineurin inhibitor (CNI) doses, were instituted in the case of the occurrence of DGF.

### 2.5. Outcomes

Complications after KT were categorized as infectious (urinary tract, cytomegalovirus, and BK virus infections), vascular (stenosis, thrombosis, or dissection of the renal artery, renal vein thrombosis, dissection and stenosis of the iliac artery, pseudoaneurysm, and hematomas), and urological (ureteral stricture and leakage, vesicoureteral reflux, urolithiasis, and urinary tract obstruction secondary to lymphocele). The treatment adopted for each complication was recorded.

After KT, before discharge, renal function (serum creatinine) and urine output were recorded for each patient. Postoperatively, we also registered the number of dialytic sessions performed during hospitalization and those executed at the peripheral center for the first year after transplantation, as well as the number of outpatient clinic visits carried out in the first 3 months after KT. Moreover, early (within 3 months after surgery) and late (4–12 months after KT) reoperations (including transplant nephrectomy), interventional radiologic procedures, and readmissions were reported. Five-year patient and graft survivals and five-year graft function were also evaluated.

According to the LOS of hospital admission for KT, patients with DGF after transplantation were divided into two groups, named the “early discharge group’, defined as patients discharged within the ERAS target (less or equal to 5 days from transplantation), and the “late discharge group”, defined as recipients discharged later (beyond 5 days after transplantation).

### 2.6. Cost Appraisal

Data related to supply costs were collected using the acquisition costs provided by the Polyclinic Tor Vergata Hospital accounting department. The cost analysis included five domains: (1) the costs of the surgical procedure (KT and eventual reoperations within the first year after KT); (2) hospital bed costs of the first hospital admission (when KT was performed) and those of eventual latter readmissions within 1 year from KT; (3) the costs of dialytic sessions occurring during the first hospital admission for KT or at the peripheral center as an outpatient during the first year after KT; (4) the costs of interventional procedures for the treatment of complications occurred during the first year after KT; (5) the costs of outpatient clinic visits in the first 3 months after KT. The variables of the costs are detailed in the Supplementary Materials (Table S1). In line with the Italian national law, a standard value-added tax (VAT) of 22% was added [29].

For the surgical procedures, we considered both fixed charges (including the costs of purchase or sterilization of surgical items) and variable overhead given by the costs for the use of the operating theater. The cost for the utilization of the operating room for 240 min, which was called the “surgical procedure time cost’, was indicated. Interventional radiology procedures’ costs included the drainage of abscess or hematoma, percutaneous nephrostomy, JJ ureteral or arterial stent placement, and kidney graft biopsy costs.

A cost analysis of the first year after KT was performed, evaluating the costs for each procedure both in patients discharged within 5 days of KT (“early discharge group”) and in those discharged late (>5 days after KT, “late discharge group”), and the results were compared between the two cohorts. Factors that should have influenced the first-year post-KT cost variations after DGF were also evaluated.

### 2.7. Statistical Analysis

Data were collected retrospectively. Statistical analyses were executed using the Statistical Package for Social Sciences (SPSS) software (Version 29.0.2.0; IBM, Chicago, IL, USA). Continuous variables were described by the median and sample minimum and maximum values, while absolute frequencies and percentages were used for categorical variables’ descriptions. The parametric Student t-test was applied in the case of normally distributed continuous variables, while the Mann–Whitney U test or Fisher exact test was used in the other cases. We used the Kaplan–Meier method to analyze time-to-event data, such as the length of hospital stay and patient and graft survival. The survival times of the two groups (i.e., early vs. delayed discharge) were compared through the log-rank test. Statistical significance was set at an alpha level of 0.05.

## 3. Results

### 3.1. Study Population

**Figure 2:**
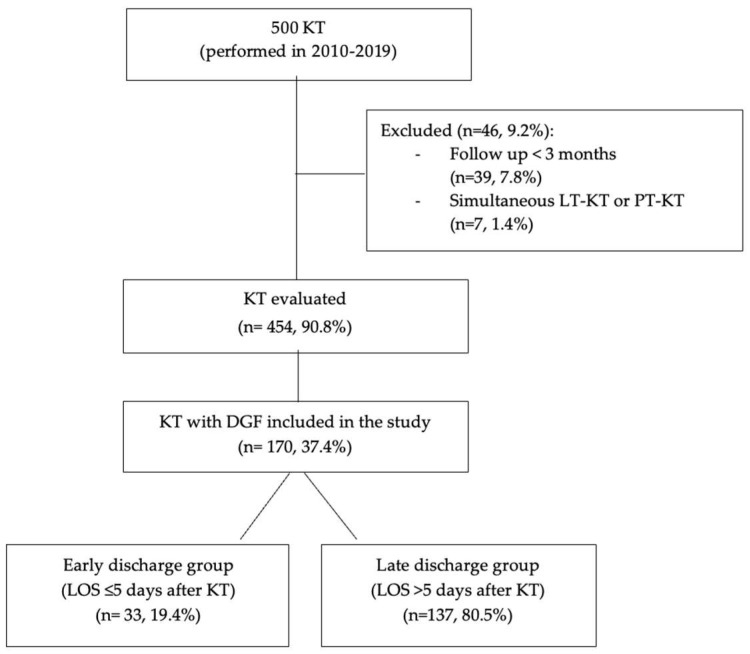
Study population. Abbreviations: kidney transplant (KT); number (n); liver transplant (LT); pancreas transplant (PT); years (y); delayed graft function (DGF); length of hospital stay (LOS).

Out of the 500 patients undergoing KT during the study period, 170 (34%) KT recipients developed DGF, and all of them had a follow-up of at least 3 months (Figure 2). Recipients, donors, and transplant characteristics are shown in Table 1.

In the entire study population, male patients were 111 (65.2%), and the median age of the recipients was 58 (25–75) years. A total of 24 (14%) patients had a BMI greater than 30, while the median BMI was 24 (16–35). Glomerulonephritis, autosomal dominant polycystic kidney disease (ADPKD), and ESKD secondary to arterial hypertension were the most representative indications for KT. At least one comorbidity was present in 83 (48.8%) recipients, namely arterial hypertension (*n* = 49, 28.8%), diabetes mellitus type 2 (*n* = 17, 10%), and cardiovascular diseases (*n* = 34, 20%).

Grafts from deceased donors after brain death were predominant (*n* = 168, 98.8%), followed by kidneys from ECD (*n* = 106, 62.3%), while the median donor age was 59 (15–83) years. A total of 163 (95.8%) recipients underwent a single transplantation, while a double KT was performed in 7 (4.1%) patients. A total of 20 (11.7%) recipients underwent a second transplant. A graft biopsy was obtained in 99 (58.2%) cases before implantation, and of those, 43 (25.2%) kidneys had a histological score > 3. The median CIT was 9 (1–29) h, with 69 (40.5%) grafts having a CIT ≥ 10 h.

When patients discharged within 5 days of transplantation (early discharge group: *n* = 33, 19.4%) were compared to those discharged after more than 5 days from KT (late discharge group: *n* = 137, 80.5%), no differences were observed in terms of recipient, donor, and transplant characteristics.

### 3.2. Postoperative Outcomes

The postoperative outcomes are reported in Table 2. The overall median follow-up was 52 (3–139) months. The median length of hospital stay for patients in the “early discharge group” was 5 (4–5) days, while the LOS was 9 (6–39) days in the “late-discharge group” (*p* < 0.001). After KT, before discharge, the two cohorts of recipients with DGF had similar renal function [serum creatinine: “early discharge group”: 8.3 (3.6–17) vs. “late discharge group”: 7.7 (1.4–16), *p =* 0.14] and similar urine output [“early discharge group”: 250 (0–2800) vs. “late discharge group”: 775 (0–4500), *p =* 0.08]. Furthermore, the “excess urine output” (i.e., the difference between urine output at discharge after KT and urine output before transplantation) was comparable between the two groups [“early discharge group”: 150 (0–2700) vs. “late discharge group”: 400 (0–3700), *p =* 0.09].

KT recipients who had a hospital stay shorter than 5 days had significantly fewer postoperative in-hospital dialysis sessions during the first hospital admission for KT than those who were discharged late after KT [2 (0–3) vs. 3 (1–9), *p* = <0.001]. Conversely, no significant differences were observed in terms of dialysis sessions at peripheral centers after discharge between the two groups [1 (0–9) vs. 1 (0–12), *p* = 0.451].

No significant differences were recorded for early (≤third-month post-KT) and late (between the fourth and twelfth month after KT) post-transplant infections, reoperations, and early interventional radiology procedure rates. Conversely, both early and late urological complications were significantly more common in the “early discharge group” than in the “late-discharge group” ([6 (18.2%) vs. 6 (4.4%) *p* = 0.013] and [7 (21.2%) vs. 9 (6.56%) *p* = 0.017]). Furthermore, late radiologic procedures (given by percutaneous nephrostomy and JJ ureteral stent placements) were more frequent in KT recipients discharged within 5 days than in those discharged late [4 (12.1%) vs. 4 (2.9%), *p* = 0.046].

Early and late readmission rates were comparable in the two groups, but patients discharged within 5 days of KT were readmitted earlier compared to those with a prolonged first hospitalization [“early discharge group”: 1 (0–3) vs. “late discharge group”: 48 (2–352), *p* <0.001]. The median number of outpatient visits after discharge [“early discharge group”: 8 (3–15) vs. “late discharge group”: 7 (2–16), *p* = 0.359] was comparable between the two groups.

**Figure 3:**
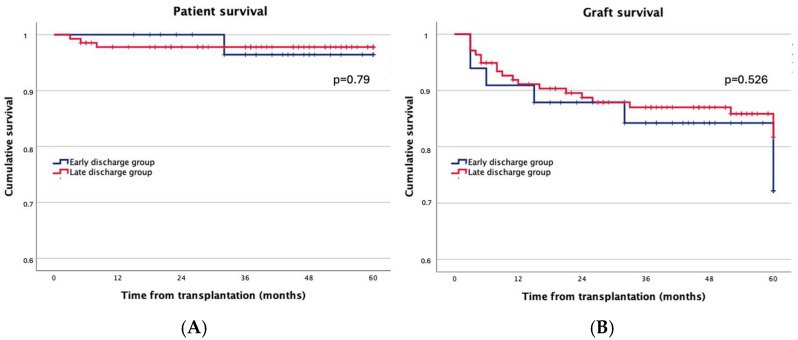
The 5-year patient survival (**A**) and 5-year graft survival (**B**) in kidney transplant recipients who developed delayed graft function. “Early discharge group” refers to patients discharged within 5 days of kidney transplantation, while the “late discharge group” comprises recipients with hospital stays longer than 5 days after transplantation. The survival times of the two groups are compared using the log-rank test. Abbreviations: enhanced recovery after surgery (ERAS).

After five years of follow-up, graft function [serum creatinine: 1.99 (0.6–13.07) mg/dL vs. 1.8 (0.68–12.0) mg/dL, *p* = 0.414], patient survival (97.0% vs. 97.8%, *p =* 0.790), and graft survival (81.8% vs. 84.7%, *p =* 0.526) were similar between the “early discharge group” and “the late discharge group” (Figure 3).

### 3.3. Cost Analysis of KT Recipients with DGF

Inpatient dialysis significantly increased the first-year post-KT costs of those recipients with DGF in the late discharge group (*p* = 0.003), while the post-discharge radiological and surgical management of complications did not significantly influence expenses one year after transplantation (*p* = 0.243 and *p* = 0.381, respectively). Outpatient management (i.e., out-of-hospital dialysis sessions and clinic visits within three months of KT) did not significantly affect the costs incurred in the first year after transplantation (*p* = 0.451 and *p* = 0.359, respectively). Overall, the total healthcare costs of the first year after KT for the “early discharge group” were significantly lower than those for the “late discharge group” [EUR 8104.76 (7218.4–28,573.2) vs. EUR 12,620.52 (8725.7–58,061.5), *p* < 0.001]. Specifically, patients in the “late discharge group” had a median excess cost (namely Δ) of EUR 4515.76 per patient.

The cost of the first hospitalization was significantly lower for KT recipients who were discharged by the fifth postoperative day after KT compared to those with a hospital stay longer than 5 days after transplantation [EUR 3870.12 (3104.12–3961.1) vs. EUR 6974.24 (4636.1–29,994.3), *p* < 0.001] with a difference resulting in a median Δ of EUR 3104.12 per patient.

After discharge for KT, no significant differences were observed between the two groups in terms of costs within the first year [EUR 228.72 (92.88–20,737.36) vs. EUR 163.96 (30.96–46,275.28), *p* = 0.207]. Details of the cost analysis are summarized in Table 3.

### 3.4. First-Year Post-KT Cost Predictors

**Figure 4:**
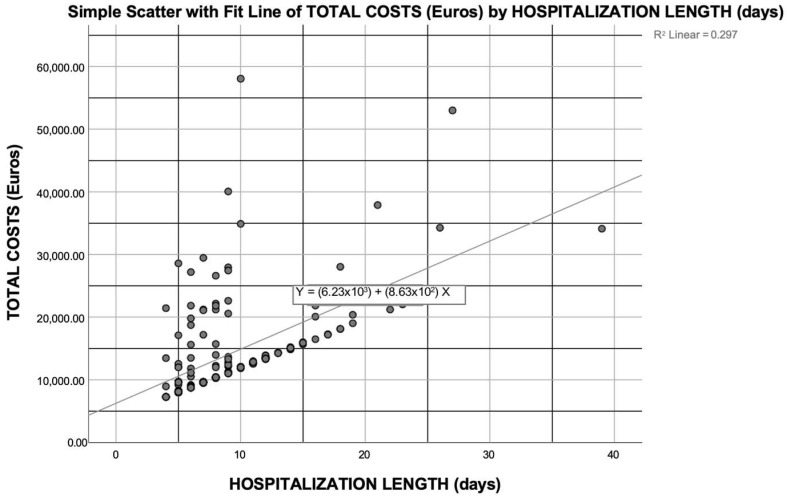
Correlation between the length of hospital stay (when kidney transplant was performed) and cost of the first year after transplantation. The term “Total costs” refers to the cost of the first year after kidney transplantation; “hospitalization length” refers to the length of the hospital stay when kidney transplantation was performed.

The costs incurred during the first hospitalization (when KT was performed) accounted for 57% of the first-year costs after transplantation, and this figure rose to 83% when the surgical costs of the first hospital admission were excluded from the cost analysis. However, different lengths of hospital stay during the first admission explained only about 29% of the variability in the costs for the first year after KT (R^2^ = 0.297), as detailed in Figure 4. Other factors that significantly affected the costs of the first year post-KT for patients experiencing DGF included the age of the recipient (*p* = 0.028) and receiving a graft from an ECD (*p* = 0.04), although these variables had a lesser impact on the costs incurred in the first year than the LOS itself (R^2^ = 0.31 and R^2^ = 0.312, respectively). Thus, none of the other recipient characteristics (BMI, gender, region of origin, and one or more comorbidities, including cardiovascular diseases and type 2 diabetes) nor CIT significantly impacted the total costs of the first year after KT.

## 4. Discussion

The present study was the first to investigate both outcomes and costs in a specific cohort of KT recipients who experienced DGF following the implementation of ERAS programs. Our findings demonstrated that achieving discharge within the “ERAS target”—five days after KT—in patients developing DGF did not compromise the early post-transplant outcomes nor the 5-year patient and graft survival. Additionally, the ERAS approach led to a significant reduction in the total costs incurred during the first year after the transplant.

DGF remains a critical and debated issue in the current transplant landscape [11]. The persistently high incidence of DGF is influenced by multiple factors, including donor-related factors (i.e., the use of ECD, which remains prevalent in our geographic and clinical context), procurement-related factors (i.e., prolonged CIT, which acts through ischemia-reperfusion injury), recipient-related factors (i.e., increasing access to KT for “frail” patients, including those with significant comorbidities, such as diabetes or cardiovascular diseases, long-standing dialysis before transplantation, or high immunological sensitization), and perioperative factors (i.e., hemodynamic instability leading to suboptimal graft reperfusion, nephrotoxic medications) [30,31]. All these risk factors could potentially overlap, lead to exacerbation of the ischemic injury, and result in DGF.

Despite advancements aimed at mitigating DGF, including the use of machine perfusion techniques, the incidence of DGF remains high, ranging from 20 to 40%, with peaks as high as 60% [30,32]. Given the significant clinical and economic impacts of DGF, optimizing strategies for managing this post-KT condition is essential. Effective management involves close monitoring of clinical status, fluid balance, and adjustments to immunosuppressive, fluid, and diuretic regimens. Furthermore, after DGF diagnosis, it is crucial to apply timely weaning from dialysis, as graft function improves, and keep vigilance regarding surgical and immunological complications after KT. Historically, in-hospital management of DGF has been the standard practice. However, prolonged hospitalization may delay surgical recovery, increase the risk of hospital-acquired infections under immunosuppressive therapy, and compromise the post-transplant quality of life. Recently, variations in DGF management practices across transplant centers—including LOS, frequency of outpatient clinic visits, and inpatient or outpatient dialysis practices—were documented by a survey across transplant centers [32]. Furthermore, well-established short-term consequences of DGF, including increased acute allograft rejection [31], can affect longer-term outcomes, such as increased graft loss, which is closely tied to the duration of DGF [33,34]. According to these data, concerns regarding the adverse impact of DGF on short- and long-term outcomes and, consequently, expected higher costs contribute to high rates of kidney offer declines when DGF risk is perceived [32].

The implementation of standardized protocols is essential in KT candidates, and it is even more significant in the case of DGF. While ERAS protocols have demonstrated significant advantages across various surgical fields, particularly colorectal surgery [1,2,3,4,5], their adoption in KT has been met with reluctance. This hesitation mainly arises from the higher surgical risks associated with KT recipients due to multiple comorbidities, higher cardiovascular-related mortality rates [6,7,8], and frail clinical status due to long-term dialysis therapy. Additional challenges in postoperative programs for KT management include the complex anesthetic management required for KT—often performed in urgent settings in cases of deceased-donor grafts—and the need for adjustments to immunosuppressive and fluid therapy, which are crucial in cases of DGF occurrence. Despite these challenges, single-center experiences with ERAS pathways in KT have yielded promising postoperative outcomes [16,17,18,19], and guidelines for implementing ERAS in KT are emerging [35].

At our center, ERAS programs in KT have been systematically applied since 2010, and we recently demonstrated that they are safe and feasible in all KT recipients [21]. Our ERAS protocols encompass four phases: pre-listing counseling, preoperative optimization, intraoperative management, and postoperative care [21]. However, the impact of the ERAS protocol on KT recipients experiencing DGF, which has been demonstrated to be a major risk factor for late discharge, had remained unexplored until now.

In the current study, our results demonstrated that the ERAS approach is both safe and effective in the specific cohort of KT recipients with DGF, without producing a significant impact on short- and long-term outcomes. Specifically, early discharge within five days after KT did not result in higher rates of reoperation (including transplant nephrectomy), readmission, and postoperative complications within the first year. The only notable exception was a higher incidence of early urological complications in patients with DGF discharged by the fifth postoperative day after transplantation. These included undiagnosed ureteral stenosis or leakages during the initial hospitalization when KT was performed, likely due to the absence of urine output at that time. Given the diagnostic challenge of detecting urinary complications in oligo-anuric patients, routinely scheduled ultrasound evaluations may aid in the identification of these events in the early post-transplant period, potentially mitigating the associated risks. Furthermore, in the present study, both the 5-year graft and -patient survival rates, as well as the 5-year graft function, were unaffected by an early discharge (within 5 days of transplantation).

KT recipients discharged within the ERAS target were managed in the outpatient setting through scheduled clinic visits. The first outpatient reassessment took place within 3 days of discharge, and follow-up visits continued regularly up until the functional recovery of the graft. According to our data, the present follow-up scheme resulted in a significant decrease in the number of inpatient dialysis sessions. This aim could be safely achieved through a close collaboration between the transplant center and the nephrologist at the patient’s dialysis center. We advocate for proactive communication with the physician to ensure seamless standardized care after transplantation. Before discharge from the hospital, the nephrologist should be informed about the transplant outcome, the immunosuppressive and diuretic regimens, and the subsequent scheduled follow-up visit appointments. After discharge, information regarding several aspects, such as ideal fluid balance targets reached after each dialysis session, optimal dry weight, or appropriate adjustments to therapy, should be regularly shared between healthcare providers. Adequate communication becomes even more relevant in cases where significant geographic distance exists between the transplant hospital and the patient’s dialysis facility. Lastly, this approach seems to facilitate the early restoration of patients’ daily routines and is usually well-perceived by the recipients.

Interestingly, the homogeneity of preoperative recipients’ characteristics (e.g., comorbidities, duration of dialysis) and donor/graft features (e.g., ECD, CIT) between patients who achieved the ERAS target of discharge (within five days of KT) and those who did not, suggests that ERAS protocols can be effectively implemented in all KT recipients with DGF. However, logistical challenges may contribute to a delayed discharge. The mentioned constraints include the need for the inpatient administration of specific antimicrobial therapies (e.g., antibiotics or antiviral agents), a long geographical distance between the transplant center and the patient’s usual dialysis facility, and being the user of a private peripheral dialysis center, where there may be limited availability of dialysis sessions on weekends and holidays. Additionally, inadequate communication between the surgeon and the nephrologist at the peripheral center regarding clinical course and follow-up scheduling after the transplant may result in the reluctance of the physician to accept the patients’ return to their usual dialysis facility. Despite these circumstances, we believe that a delayed discharge strategy, which has been traditionally adopted in the case of DGF, lacks clinical justification and is not supported by evidence. The traditional practice of prolonging the hospitalization of this specific cohort of KT recipients should be re-evaluated in favor of evidence-based patient-centered care strategies. Incorporating ERAS protocols into all phases of KT care, starting from the patient’s counseling in the preoperative period, is critical to their implementation [36].

Healthcare cost reduction remains a cornerstone of ERAS protocols and has been well-documented in various surgical specialties [37,38,39]. In the field of KT, cost analysis is a critical topic, particularly when considering the economic burden of dialysis versus transplantation. Previous studies have shown that the cost savings associated with KT are already evident within the first year after KT compared to continuing dialysis [40,41]. However, the economic issue of KT is multifaceted, influenced by factors such as reimbursement models (e.g., diagnosis-related groups), local cost indices, and different transplant center strategies [42]. For example, an increased number of candidates with multiple comorbidities on the waiting list can elevate costs if not offset by an adequate number of performed transplants due to low graft availability [42]. Strategies like promoting LDKT and minimizing the discard of grafts at risk of complications, such as DGF, are essential for cost containment. When analyzing the specific subgroup of KT recipients who experience DGF, studies consistently show that DGF significantly increases the overall costs of KT compared to the cases with immediate graft function. This cost disparity is evident when evaluating the costs incurred during both the initial hospitalization [22] and the first 90 days after KT [23]. The prolonged LOS and the surgical costs, including KT and potential reoperations, account for approximately one-third of the total annual costs [19,21,43].

Our findings indicate that the first hospitalization (when KT is performed) accounts for 57% of the total first-year costs in the case of DGF. When surgical costs are excluded, this figure rises to 83%, underscoring the critical impact of the initial admission. Although the LOS during the first hospitalization (mainly due to hospital bed costs) is a major driver of costs, our analysis shows that it explains only about 30% of cost variability. Thus, 70% of the cost changes are influenced by other factors, such as inpatient dialysis and postoperative complications, as claimed in the literature [44]. According to our data, inpatient dialysis significantly increased the first-year post-KT costs in patients with prolonged hospitalization after DGF, while post-discharge radiological or surgical management of complications did not have a statistically significant impact on the costs one year after transplantation. Moreover, our study demonstrated that outpatient management, including out-of-hospital dialysis and clinic visits within three months of KT, did not significantly affect costs. Lastly, prolonged hospitalization beyond five days after KT was associated with a median increase of EUR 3104/patient for the first hospitalization and EUR 4515/patient for the first year post-KT.

Recipient age and donor type (e.g., ECD) were also significant factors influencing the first-year costs of KT, although their impact was less pronounced than that of LOS. However, we believe that the traditional practice of prolonging the hospital stay for older recipients or those receiving ECD grafts appears unwarranted, as it is not associated with better outcomes. Variability in clinical management between transplant centers also contributes to cost differences. Factors such as graft biopsy rates, laboratory diagnostics, and imaging studies vary widely and can significantly influence first-year post-KT costs [44]. In this context, standardizing DGF management protocols seems essential to addressing this variability.

This study has some limitations, including its retrospective nature and single-center design. Furthermore, we decided to avoid comparison with an old cohort of KT recipients before 2010 (when ERAS approaches were integrated into our routine clinical practice for KT management) because of clear differences in terms of aspects such as immunosuppressive regimens and surgical technique compared to those who received a transplant within the last decade at our center. Furthermore, given the reimbursement model currently used in the Italian healthcare system (i.e., Diagnosis-Related Groups (DRG), the results of the cost analysis in the present study may not fit perfectly with different reimbursement models applied elsewhere. Lastly, while the conducted analysis accounted for major cost drivers, other indirect costs, such as long-term economic impacts, were not included.

## 5. Conclusions

Although DGF remains a complex challenge in KT management, our findings highlight the safety and cost-effectiveness of the ERAS approach in this context. Early discharge within five days of KT, even for recipients experiencing DGF, reduces healthcare costs without compromising clinical outcomes. Future research should focus on strategies for customizing specific protocols for KT recipients who develop DGF, further refining postoperative management strategies in these subjects. This approach may potentially lead to the maximization of graft utilization, along with economic savings. Standardizing care protocols and supporting healthcare providers with robust data will be critical to optimize outcomes in this specific KT recipient cohort.

## Figures and Tables

**Table 1:** Characteristics of kidney transplant recipients with delayed graft function undergoing “enhanced recovery after surgery” (ERAS) protocol.

Variables	Overall Population (n = 170)	Early Discharge Group (n = 33, 19.4%)	Late Discharge Group (n = 137, 80.5%)	*p* Value
	n (%) or Median (Sample Min–Max)	n (%) or Median (Sample Min–Max)	n (%) or Median (Sample Min–Max)	
**Recipient**				
Age (years)	58 (25–75)	54 (34–75)	63 (29–74)	0.162
Age > 60 years	81 (47.6%)	12 (36.3%)	69 (50.4%)	0.176
Gender (male)	111 (65.2%)	23 (69.7%)	88 (64.2%)	0.685
BMI	24 (16–35)	24 (17–35)	25 (18–32)	0.673
Obesity (BMI ≥30)	24 (14.1%)	7 (21.2%)	17 (12.4%)	0.262
Cause of ESKD:				0.598
-Glomerulonephritis	58 (34.1%)	15 (45.5%)	43 (31.2%)	
-ADPKD	40 (23.5%)	7 (21.2%)	33 (24.1%)	
-Arterial hypertension	19 (11.1%)	2 (6.1%)	17 (12.4%)	
-Pyelonephritis	10 (5.8%)	2 (6.1%)	8 (5.8%)	
-Unknown ESRD	14 (8.2%)	4 (12.1%)	10 (7.3%)	
-Diabetes	12 (7.06%)	2 (6.1%)	10 (7.3%)	
-Congenital malformation	4 (2.3%)	0 (0%)	4 (2.9%)	
-Other causes *	13 (7.6%)	1 (3%)	12 (8.8%)	
**Median time on waiting list (days)**	807 (13–3821)	988 (83–2816)	611 (13–3185)	0.152
Comorbidities:	83 (48.8%)	12 (36.3%)	71 (51.8%)	0.124
-Arterial hypertension	49 (28.8%)	6 (18.1%)	43 (35.5%)	0.198
-Cardiovascular diseases	34 (20%)	4 (12.1%)	30 (21.9%)	0.237
-DMII	17 (10%)	5 (15.1%)	12 (8.8%)	0.329
**-Comorbidities ≥ 2**	12 (7.05%)	2 (6.06%)	10 (7.3%)	1.000
**Donor**				
Type of donor:				1.000
-Brain dead donor	168 (98.8%)	33 (100%)	135 (98.5%)	
-Living donor	2 (1.2%)	0 (0%)	2 (1.5%)	
Age (years)	59 (15–83)	54 (34–76)	62 (27–83)	0.289
Age > 60 years	82 (48.2%)	12(36.4%)	70 (51.1%)	0.174
Cause of death:				0.298
-Cerebral hemorrhage	108 (63.5%)	21 (63.6%)	87 (63.5%)	
-Head trauma	33 (19.4%)	3 (9.1%)	30 (21.9%)	
-Ischemic stroke	15 (8.8%)	5 (15.2%)	10 (7.3%)	
-Anoxic encephalopathy	10 (5.8%)	3 (9.1%)	7 (5.1%)	
-Other causes	4 (2.3%)	1 (3%)	3 (2.2%)	
Comorbidities:				
-Cardiovascular disease	41 (24.1%)	9 (27.3%)	32 (23.4%)	0.654
-Arterial hypertension	76 (44.7%)	17 (51.5%)	59 (43.1%)	0.437
**-≥2 comorbidities**	33 (19.4%)	5 (15.2%)	28 (20.4%)	0.627
**ECD**	106 (62.3%)	19 (57.6%)	87 (63.5%)	0.553
**Transplant**				
Type of KT:				0.186
-Single KT	163 (95.8%)	33(100%)	130 (94.9%)	
-Dual KT: unilateral/bilateral	7 (4.1%): 4 (2.3%)/3 (1.7%)	0 (0%)	7 (5.1%) 4 (2.9%)/3 (2.2%)	
Re-transplant	20 (11.7%)	6 (18.2%)	14 (10.2%)	0.204
Sequential KT after LT	1 (0.5%)	0 (0%)	1 (0.7%)	0.624
Pre-implant renal biopsy:	99 (58.2%)	19 (57.6%)	80 (58.4%)	0.932
-Renal biopsy score ≤ 3	56 (32.9%)	9 (27.2%)	47 (34.3%)	0.443
-Renal biopsy score > 3	43 (25.2%)	10 (3.04%)	33(24.08%)	0.371
Median CIT (hours)	9 (1–29	8.3 (2.06–15.5)	9.6 (1–23)	0.060
CIT ≥ 10 h	69 (40.5%)	11 (33.3%)	58 (42.3%)	0.431

“Early discharge group” refers to patients discharged within 5 days of kidney transplantation, while “late discharge group” comprises recipients with hospital stays longer than 5 days after transplantation. * Other causes of ESKT include unspecified, SLE, vasculitis, HUS, drug-induced nephropathy, cystinosis, and oxalosis. Abbreviations: kidney transplantation (KT); body mass index (BMI); autosomal dominant polycystic kidney disease (ADPKD); end-stage kidney disease (ESKD); systemic lupus erythematosus (SLE); hemolytic uremic syndrome (HUS); diabetes mellitus type II (DMII); expanded-criteria donor (ECD); liver transplantation (LT); cold ischemia time (CIT); number (n); minimum (min); maximum (max); and enhanced recovery after surgery (ERAS).

**Table 2:** Outcomes of kidney transplant recipients with delayed graft function undergoing “enhanced recovery after surgery” (ERAS) protocol.

Variables	Overall Population (n = 170)	Early Discharge Group (n = 33, 19.4%)	Late Discharge Group (n = 137, 80.5%)	*p* Value
	n (%) or Median (Sample Min–Max)	n (%) or Median (Sample Min–Max)	n (%) or Median (Sample Min–Max)	
**Outcomes**				
LOS (days)	8 (4–39)	5 (4–5)	9 (6–39)	*<0.001*
Postoperative in-hospital dialytic sessions during first hospitalization	3 (1–9)	2 (0–3)	3 (1–9)	*<0.001*
KT recipients who had a single postoperative (in-hospital) dialysis session within 3 months after KT	20 (11.7%)	7 (21%)	13 (9.7%)	0.07
Postoperative dialytic sessions at peripheral center within 3 months after discharge	1 (0–12)	1 (0–9)	1 (0–12)	0.451
KT recipients who had ≤4 dialysis sessions at peripheral center within 3 months after KT	161 (94.7%)	31 (93%)	130 (97.7%)	0.258
Early complications (≤3 months):	52 (30.5%)	11 (33.3%)	41 (29.9%)	0.680
-Infectious	46 (27%)	8 (24.2%)	38 (27.7%)	0.828
-Urological	12 (7%)	6 (18.2%)	6 (4.4%)	*0.013*
-Vascular	0 (0%)	0 (0%)	0 (0%)	-
Late complications (4–12 months):	66 (38.8%)	14 (42.4%)	52 (37.9%)	0.692
-Infectious	59(34.7%)	10 (30.3%)	49 (35.7%)	0.685
-Urological	16 (9.4%)	7 (21.2%)	9 (6.56%)	*0.017*
-Vascular	0 (0%)	0 (0%)	0 (0%)	1
Reoperations (after KT):				
-Early (≤3 months)	2 (1.1%)	1 (3%)	1 (0.7%)	0.351
-Late (4–12 months)	0 (0%)	0 (0%)	0 (0%)	1
Transplant nephrectomy:				
-Early (≤3 months)	1 (0.5%)	0 (0%)	1 (0.7%)	1
-Late (4–12 months)	3 (1.7%)	1 (3%)	2 (1.4%)	0.479
Interventional radiology procedures:				
Early (≤3 months):	8 (4.7%)	1 (3%)	7 (5.1%)	1
- Percutaneous drainage of fluid (abscess, hematoma)	5 (2.9%)	0 (0%)	5 (3.6%)	0.584
- Percutaneous nephrostomy and JJ ureteral stent placement	4 (2.3%)	1 (3%)	3 (2.1%)	0.582
Late (4–12 months)	8 (4.7%)	4 (12.1%)	4 (2.9%)	*0.046*
- Percutaneous drainage of fluid (abscess, hematoma)	0 (0%)	0 (0%)	0 (0%)	1
- Percutaneous nephrostomy and JJ ureteral stent placement	8 (4.7%)	4 (12.1%)	4 (2.9%)	*0.046*
- Angioplasty and stent	0 (0%)	0 (0%)	0 (0%)	1
**Readmission rates after KT:**	79 (46.4%)	10 (30.3%)	69 (50.3%)	0.051
-Early (≤3 months)	61 (35.8%)	9 (27.2%)	52 (38%)	0.313
-Late (4–12 months)	18 (10.5%)	1 (3%)	17 (12.4%)	0.203
Time of readmission since KT (days)	8 (1–352)	1 (0–3)	48 (2–352)	*<0.001*
Outpatient clinic reviews within 3 months after KT	7 (2–16)	8 (3–15)	7 (2–16)	0.359

“Early discharge group” refers to patients discharged within 5 days after kidney transplantation, while the “late discharge group” comprises recipients with hospital stays longer than 5 days after transplantation. *p* values < 0.05 are shown in italics. Abbreviations: kidney transplantation (KT); length of hospital stay (LOS); enhanced recovery after surgery (ERAS); number (n); minimum (min); and maximum (max).

**Table 3:** Cost analysis of the first year after kidney transplantation.

Cost Variables	Early Discharge Group (n = 33, 19.4%)	Late Discharge Group (n = 137, 80.5%)		
	Median (Sample Min–Max)	Median (Sample Min–Max)	Δ	*p* Value
**Costs of KT hospital admission**	EUR 3870.12 (3104.12–3961.1)	EUR 6974.24 (4636.1–29,994.3)	EUR 3104.12	*<0.001*
1. Surgical items plus procedure time (KT) costs	EUR 3965.8 (3965.8–3965.8)	EUR 3965.8 (3965.8–3965.8)	EUR 0	1
2. Hospital bed costs	EUR 3830 (3064–3830)	EUR 6894 (4596–29,874)	EUR 3064	*<0.001*
3. In-hospital dialysis session costs	EUR 80.24 (40.12–120.36)	EUR 120.36 (40.12–361.08)	EUR 40.12	*<0.001*
4. Interventional radiology procedure costs	EUR 0 (0–508.32)	EUR 0 (0–508.32)	EUR 0	0.293
**Costs incurred after hospital admission for KT within one year of KT**	EUR 228.72 (92.88–20,737.36)	EUR 163.96 (30.96–46,275.28)	EUR −64.76	0.207
1. Surgical items plus procedure time (KT, transplant nephrectomy, surgical revisions) costs	EUR 0 (0–3947.8)	EUR 0 (0–3947.8)	EUR 0	0.381
2. Hospital bed costs	EUR 0 (0–19,916)	EUR 0 (0–45,960)	EUR 0	0.886
3. In-hospital dialysis session costs	EUR 0 (0–641.92)	EUR 0 (320.96)	EUR 0	0.076
4. Outpatient dialysis session costs	EUR 40.12 (0–361.08)	EUR 40.12 (481.44))	EUR 0	0.451
5. Interventional radiology procedure costs	EUR 0 (0–1485.12)	EUR 0 (0–2473.32)	EUR 0	0.414
6. Costs of outpatient clinic reviews within 3 months	EUR 123.8 (46.4–232.2)	EUR 108.36 (30.9–247.6)	EUR −16	0.359
**Total costs of the first year after KT**	EUR 8104.76 (7218.4–28,573.2)	EUR 12,620.52 (8725.7–58,061.5)	EUR 4515.76	*<0.001*
1. Surgical items plus procedure time (KT, transplant nephrectomy, surgical revisions) costs	EUR 3965.8 (3965.8–7913.8)	EUR 3965.8 (3965.8–7913.8)	EUR 0	0.381
2. Hospital bed costs	EUR 3830 (3064–23,746)	EUR 8426 (4596–53,620)	EUR 4596	*<0.001*
3. In-hospital dialysis session costs	EUR 80.24 (40.12–682.04)	EUR 120.36 (40.12–481.44)	EUR 40.12	*0.003*
4. Outpatient dialysis session costs	EUR 40.12 (0–361.08)	EUR 40.12 (0–481.44)	EUR 0	0.451
5. Interventional radiology procedure costs	EUR 0 (0–1485.12)	EUR 0 (0–2473.32)	EUR 0	0.243
6. Outpatient dialysis session costs	EUR 40.12 (0–361.08)	EUR 40.12 (0–481.44)	EUR 0	0.451

“Early discharge group” refers to patients discharged within 5 days of kidney transplantation, while the “late discharge group” comprises recipients with hospital stays longer than 5 days after transplantation. *p* values < 0.05 are in italic characters. Abbreviations: kidney transplantation (KT); euros (EUR); number (n); minimum (min); maximum (max); and the difference between the median of the costs of patients discharged within 5 days of KT and the median of the costs of recipients discharged after more than 5 days from KT (Δ).

## Data Availability

The data reported in the study are available upon request from the corresponding author. Due to confidentiality, data are not publicly accessible.

## Supplementary Materials

The following supporting information can be downloaded at: https://www.mdpi.com/article/10.3390/jcm14072387/s1, Table S1: Healthcare costs for kidney transplant recipients.

## Abbreviations

The following abbreviations are used in this manuscript:

ERAS	Enhanced recovery after surgery
KT	Kidney transplantation
DGF	Delayed graft function
LOS	Length of hospital stay
ECDs	Expanded-criteria donors
BMI	Body mass index
ESKD	End-stage kidney disease
CIT	Cold ischemia time
PRA	Panel-reactive antibody
CNIs	Calcineurin inhibitors
VAT	Value-added tax
ADPKD	Autosomal dominant polycystic kidney disease
EUR	Euros
