# Supplementary material for: The Enhanced Recovery After Surgery Pathway Is Safe, Feasible and Cost-Effective in Delayed Graft Function After Kidney Transplant
Source: J Clin Med. 2025 Mar 31;14(7):2387. doi: 10.3390/jcm14072387 (PMC11990043; doi:10.3390/jcm14072387)
Supplement: Supplementary file 1 [file jcm-14-02387-s001.zip › jcm-3488613-supplementary.pdf]

SUPPLEMENTARY MATERIALS

Table S1. Healthcare costs for kidney transplant recipients

		COSTS (€)
**1.**	**SURGICAL PROCEDURE (KT / transplant nephrectomy / surgical revision)**	
-	SURGICAL DISPOSABLE ITEMS	
Multiple-use materials:		
•	Surgical kidney transplantation kit	240
•	Surgical retractor Omnitract ® (Integra, USA)	240
Single use items:		
•	Double J ureteral stent	27
•	Steri-drape	18
•	Argon plasma coagulation	134
•	Suction tube	5.8
•	Intra- and post-operatory devices and kits for blood collection, washing and reinfusion	901
-	SURGICAL PROCEDURE TIME: 600 €/h (average time for KT: 4 hours)	2400
**2.**	**HOSPITAL BED (per day)**	766
**3.**	**SINGLE HAEMODIALYSIS SESSION ITEMS**	40.12
•	Bicarbonate bag	3.5
•	Filter	15
•	Infusion line	3.64
•	Arterial/vein line	11
•	Fistula connection/disconnection kit	2
•	2 fistula needles (0.41 each)	0.82
•	Acid concentrate bag	4.16
**4.**	**INTERVENTIONAL RADIOLOGY PROCEDURES ITEMS**	
-	Percutaneous nephrostomy/percutaneous drainage of fluid (abscess, hematoma)	508.32
•	Single-use percutaneous drainage for nefrostomy	170.8
•	Hydrophilic guidewires for catheterism	79.3
•	Vascular dilators (at least 4 Fr diameter)	10.86
•	Set for "access": puncture needle 21/22 Ga, cannula, sheath, dilator	134.2
•	Hydrophilic angiographic selective catheters (at least 4 Fr, polyurethane).	109.8
•	Sterile drape	3.36
-	Double J ureteral stent placement	976.8
•	Single-use percutaneous drainage for nefrostomy	170.8
•	Hydrophilic guidewires for catheterism	79.3
•	Vascular dilators (at least 4 Fr diameter)	10.86
•	Full set for "access": puncture needle 21/22 Ga, cannula, sheath, dilator	134.2
•	Hydrophilic angiographic selective catheters (at least 4 Fr, polyurethane	109.8
•	Ureteral stent	176.9
•	Metallic guidewires teflon-coated	84.18
•	Vascular sheats (4 Fr diameter, 10 cm lenght)	13.42
•	Balloon catheter for angioplasty	193.98
•	Sterile drape	3.36
-	Angiography/angioplasty/arterial stent placement	2422.38
•	Alloy metal/PTFE graft stents	1798.16
•	Hydrophilic guidewires for catheterism	183
•	Support microcatheter	280.6
•	Sheath set	132.98
•	Vascular sheats (4 Fr diameter, 10 cm length)	13.42
•	Vascular dilators (at least 4Fr diameter)	10.86
•	Sterile drape	3.36
-Percutaneous kidney graft biopsy		50.94
•	Automatic needle for percutaneous biopsy	47.58
•	Sterile drape	3.36
**5.**	**OUTPATIENT CLINIC SINGLE REVIEW**	15.48

Abbreviations: Kidney transplant (KT); euros (€); French (Fr); Gauge (Ga); centimeters (cm); Polytetrafluoroethylene (PTFE)
